# Supplementary figures and images for: Oncogenic and teratogenic effects of Trp53Y217C, an inflammation-prone mouse model of the human hotspot mutant TP53Y220C
Source: eLife. 2025 Apr 14;13:RP102434. doi: 10.7554/eLife.102434 (PMC11996178; doi:10.7554/eLife.102434)

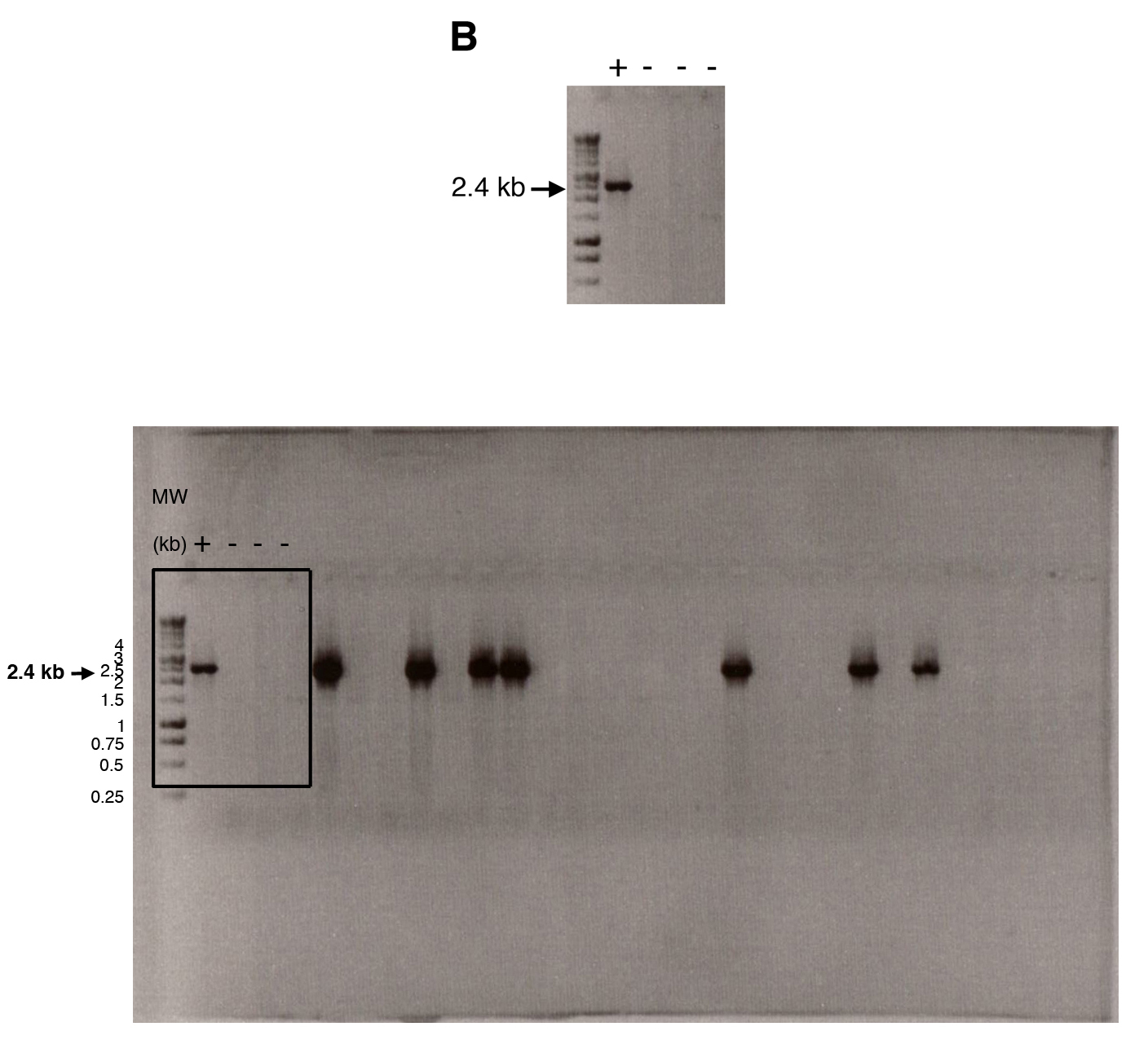

Supplement: Figure 1—source data 1. [file elife-102434-fig1-data1.zip › Fig1-sourcedata1/Jaber-sourcedata1-Fig1B.jpg]

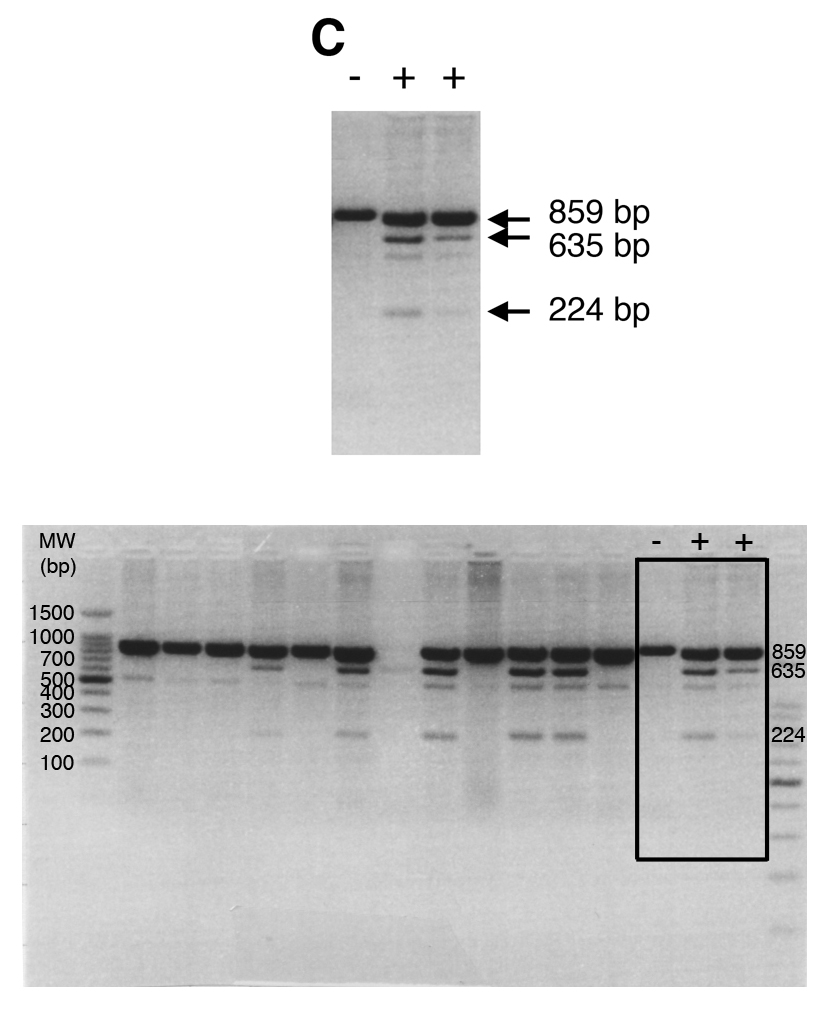

Supplement: Figure 1—source data 1. [file elife-102434-fig1-data1.zip › Fig1-sourcedata1/Jaber-sourcedata1-Fig1C.jpg]

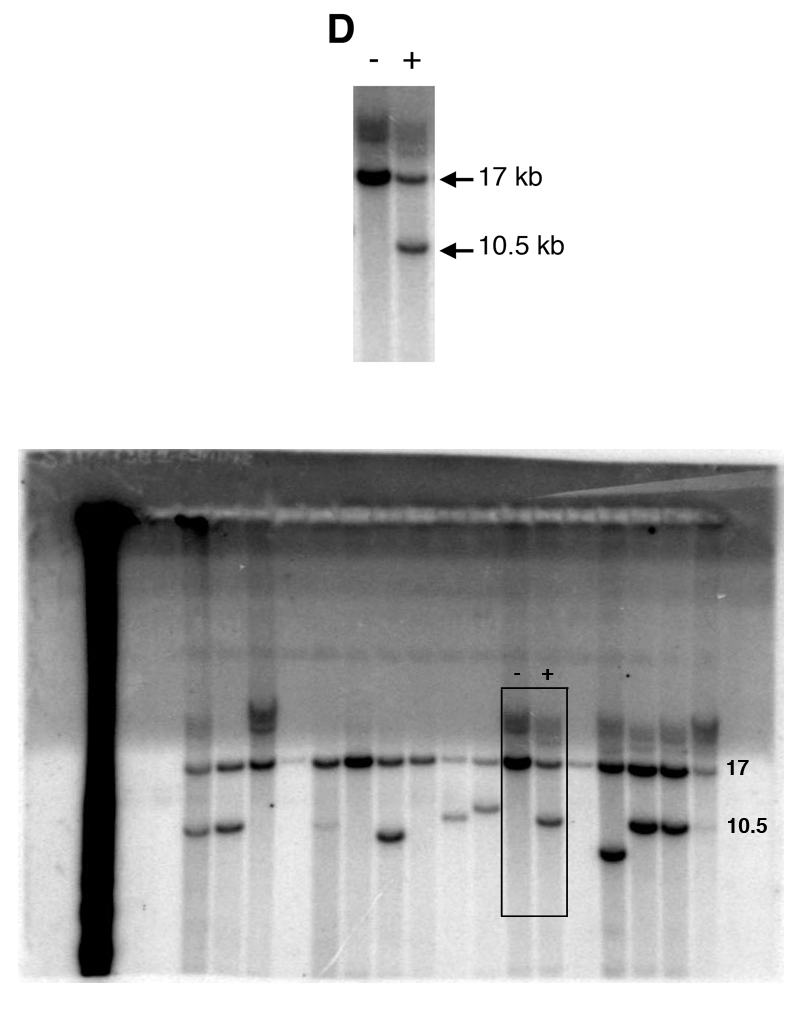

Supplement: Figure 1—source data 1. [file elife-102434-fig1-data1.zip › Fig1-sourcedata1/Jaber-sourcedata1-Fig1D.jpg]

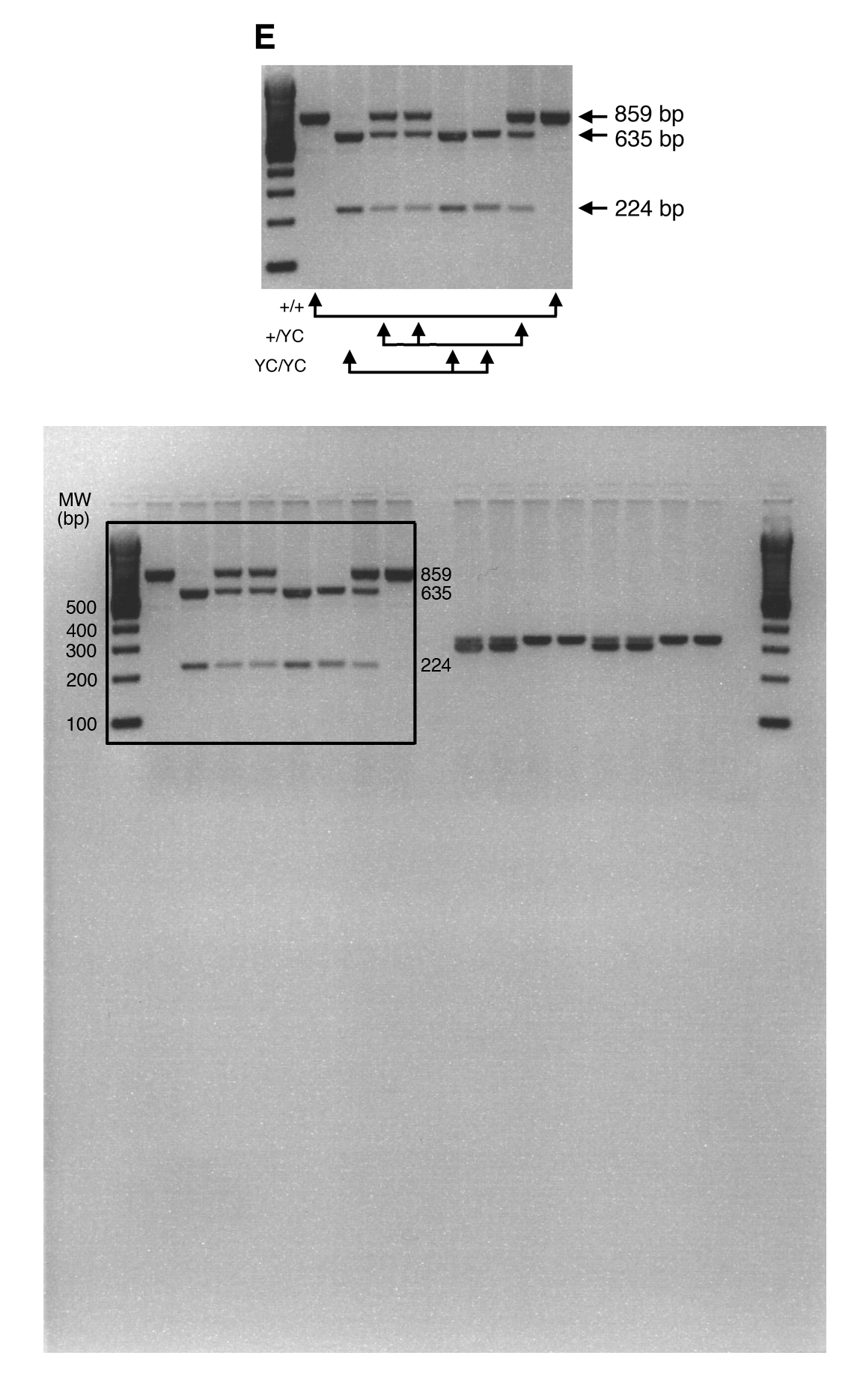

Supplement: Figure 1—source data 1. [file elife-102434-fig1-data1.zip › Fig1-sourcedata1/Jaber-sourcedata1-Fig1E.jpg]

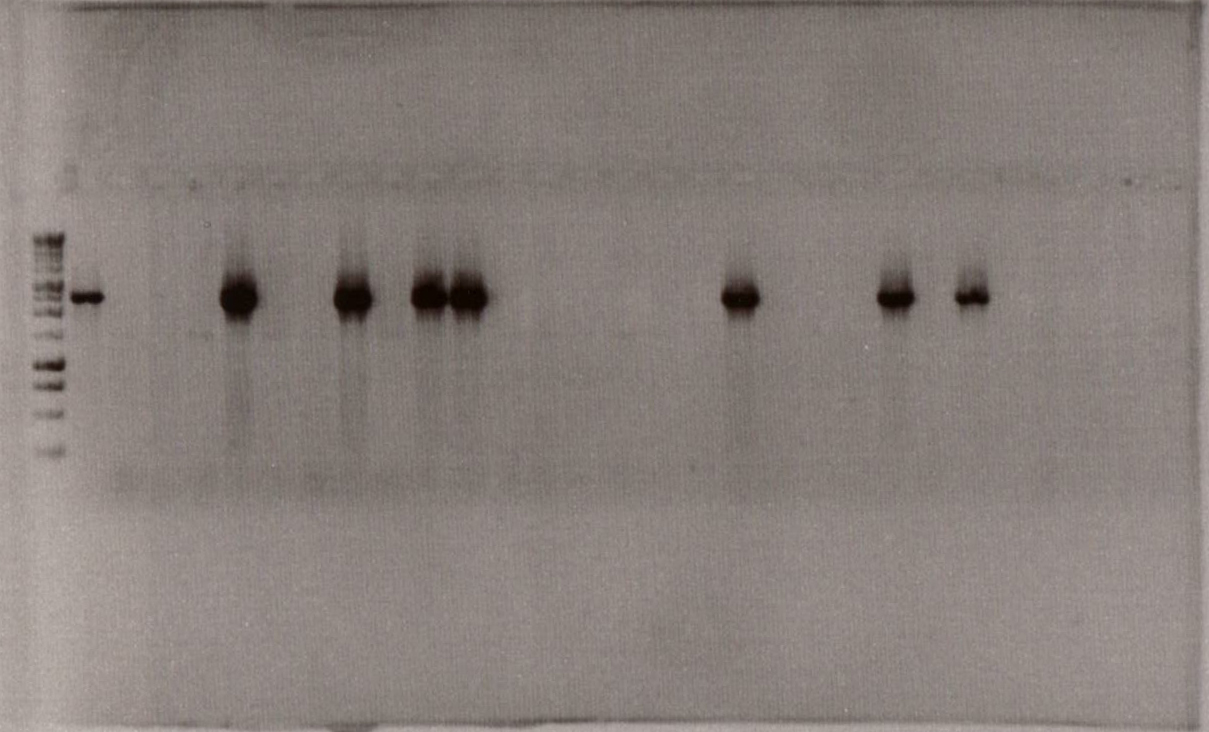

Supplement: Figure 1—source data 2. [file elife-102434-fig1-data2.zip › Fig1-sourcedata2/Jaber-sourcedata2-Fig1B.jpg]

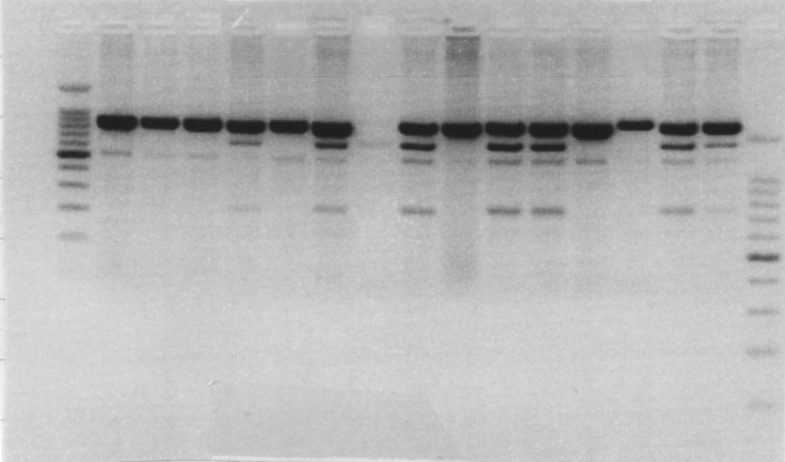

Supplement: Figure 1—source data 2. [file elife-102434-fig1-data2.zip › Fig1-sourcedata2/Jaber-sourcedata2-Fig1C.jpg]

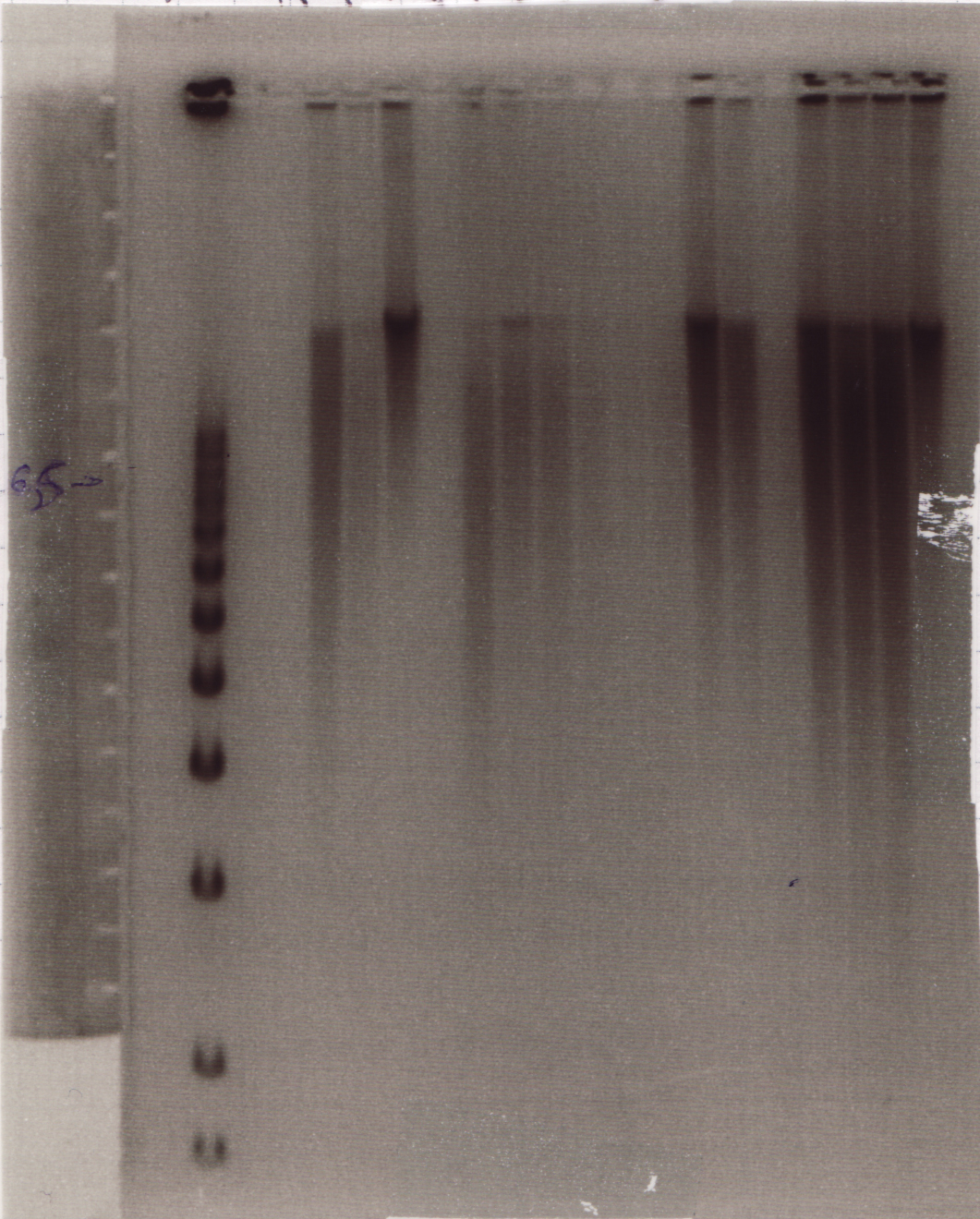

Supplement: Figure 1—source data 2. [file elife-102434-fig1-data2.zip › Fig1-sourcedata2/Jaber-sourcedata2-Fig1D-ladder.jpg]

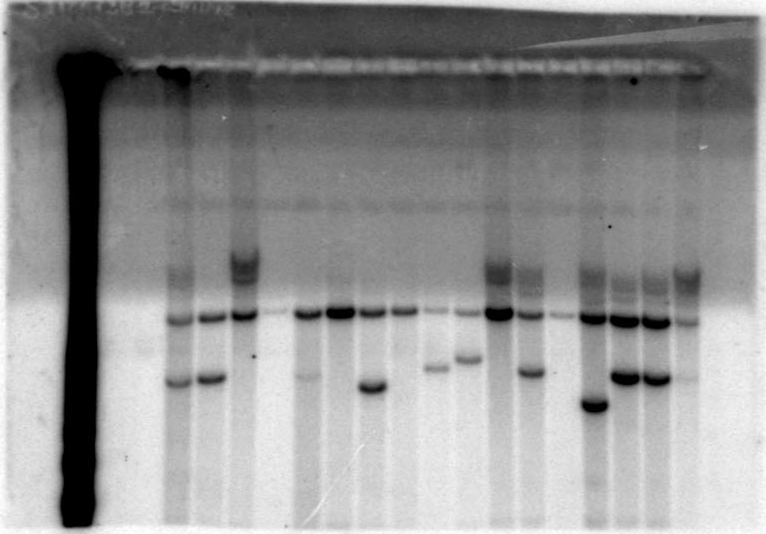

Supplement: Figure 1—source data 2. [file elife-102434-fig1-data2.zip › Fig1-sourcedata2/Jaber-sourcedata2-Fig1D-Southern.jpg]

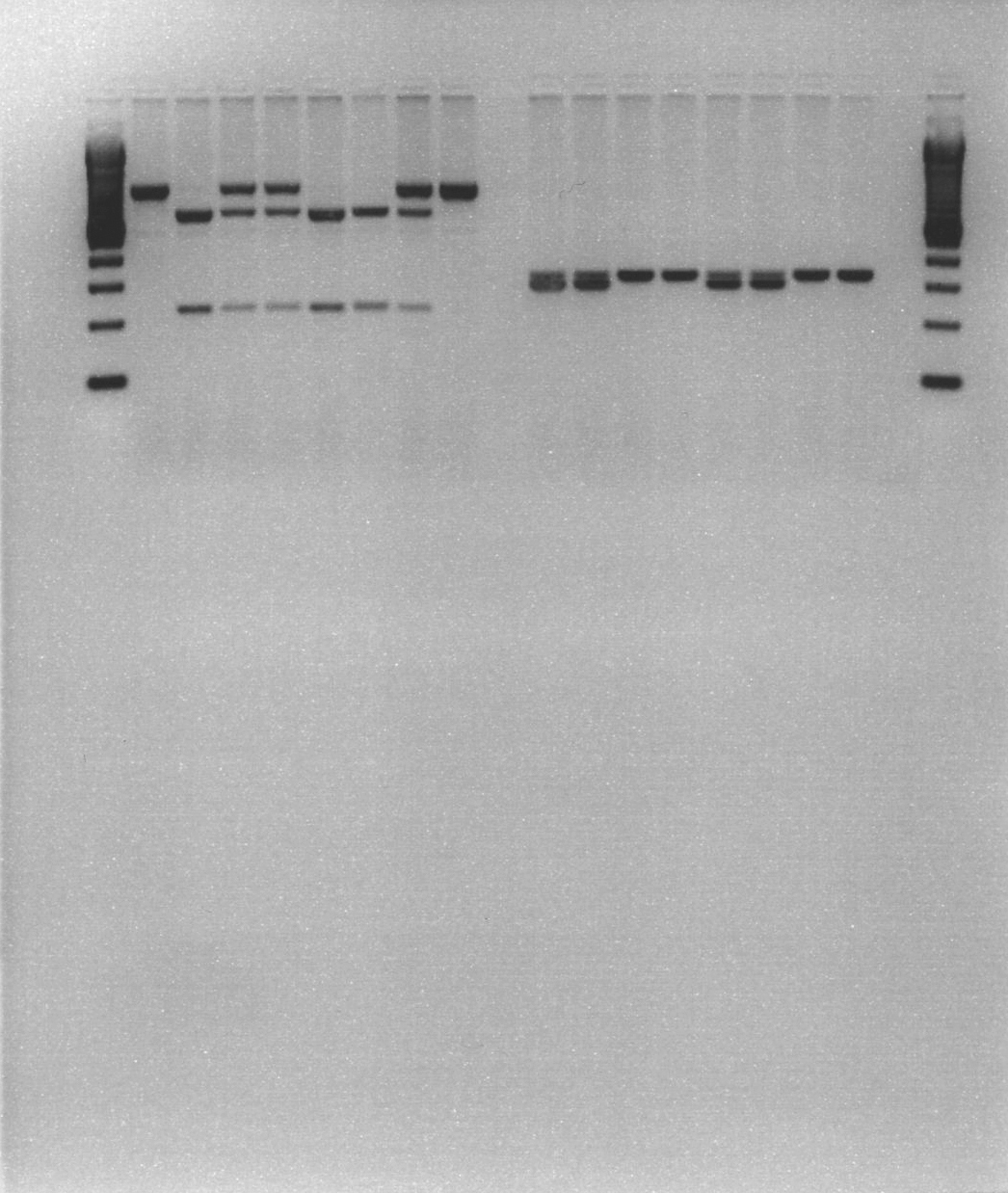

Supplement: Figure 1—source data 2. [file elife-102434-fig1-data2.zip › Fig1-sourcedata2/Jaber-sourcedata2-Fig1E.jpg]

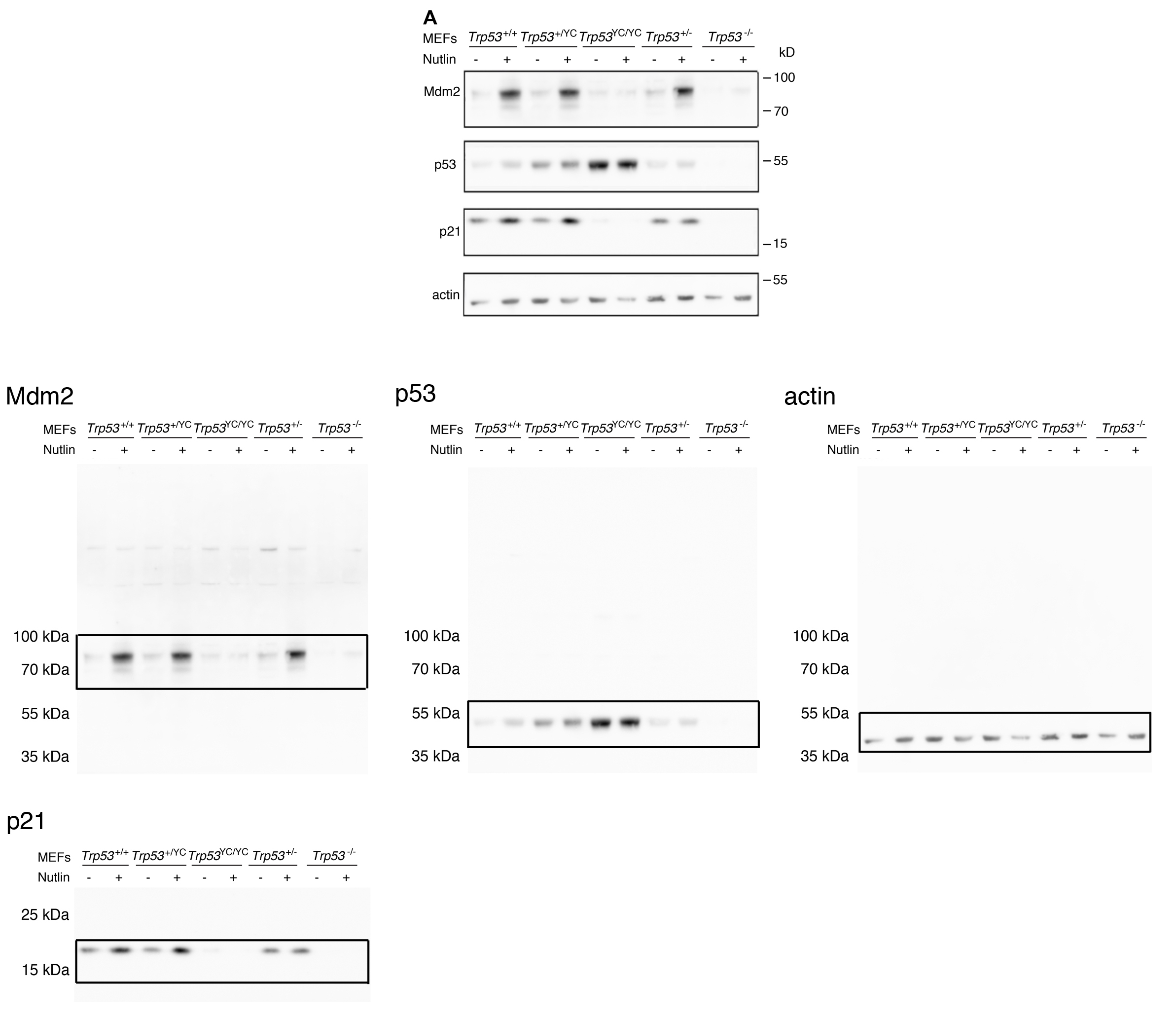

Supplement: Figure 2—source data 1. [file elife-102434-fig2-data1.zip › Fig2-sourcedata1/Jaber-sourcedata1-Fig2A.jpg]

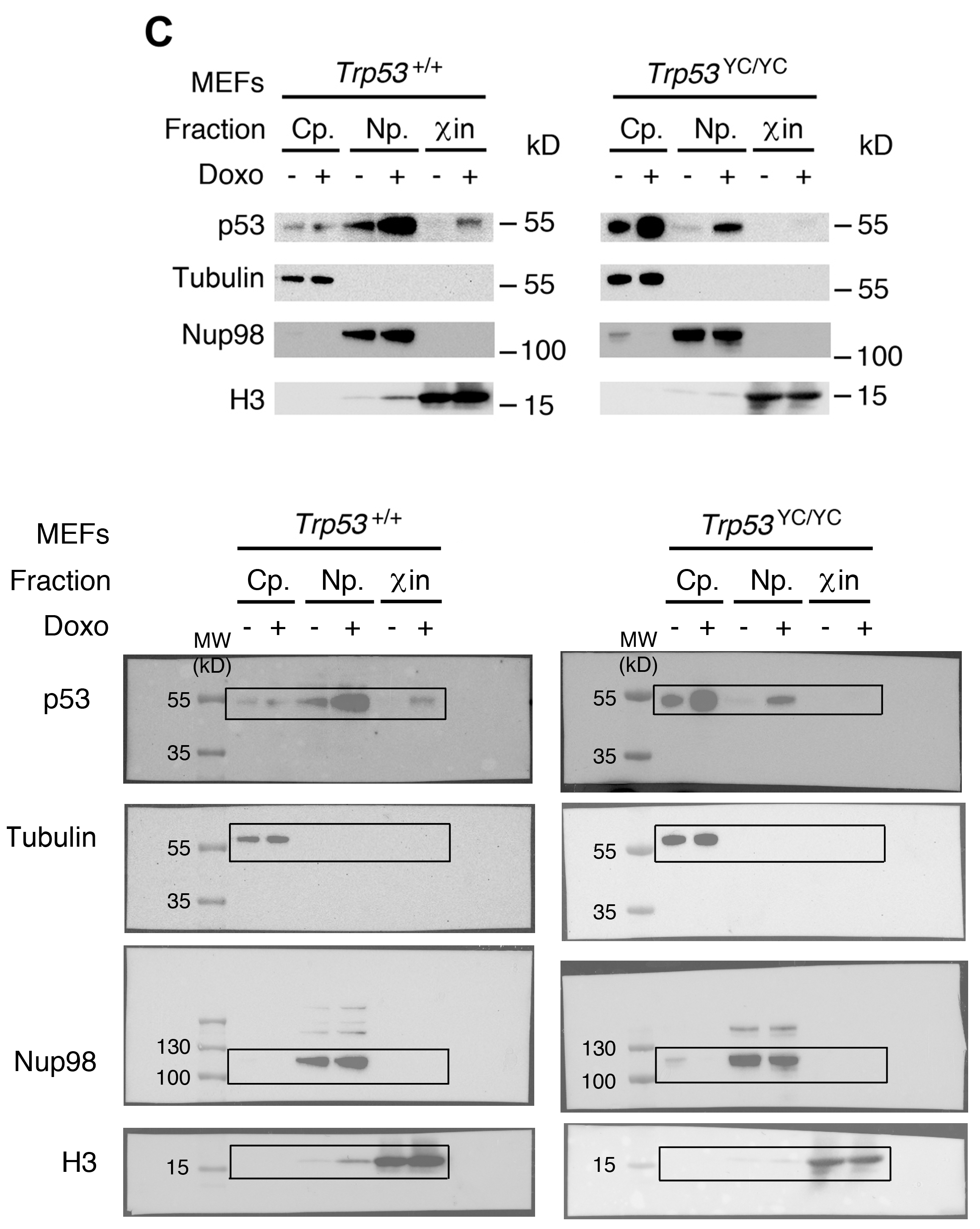

Supplement: Figure 2—source data 1. [file elife-102434-fig2-data1.zip › Fig2-sourcedata1/Jaber-sourcedata1-Fig2C.jpg]

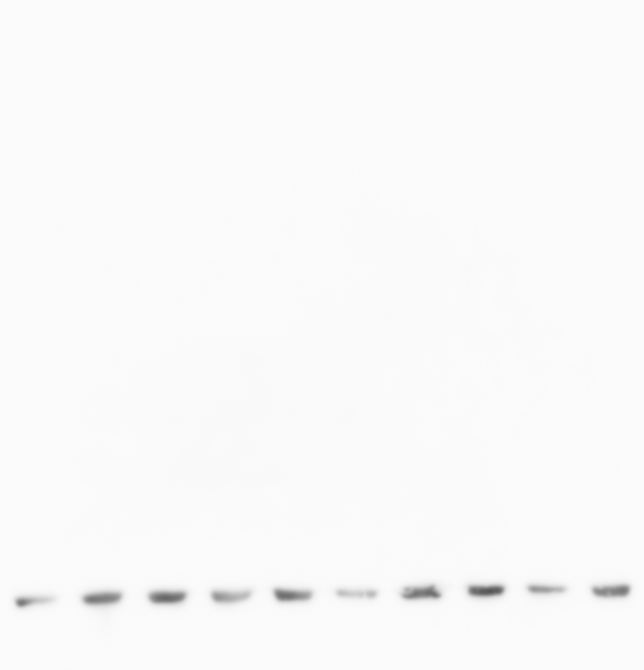

Supplement: Figure 2—source data 2. [file elife-102434-fig2-data2.zip › Fig2-sourcedata2/Jaber-sourcedata2-Fig2A-actin.jpg]

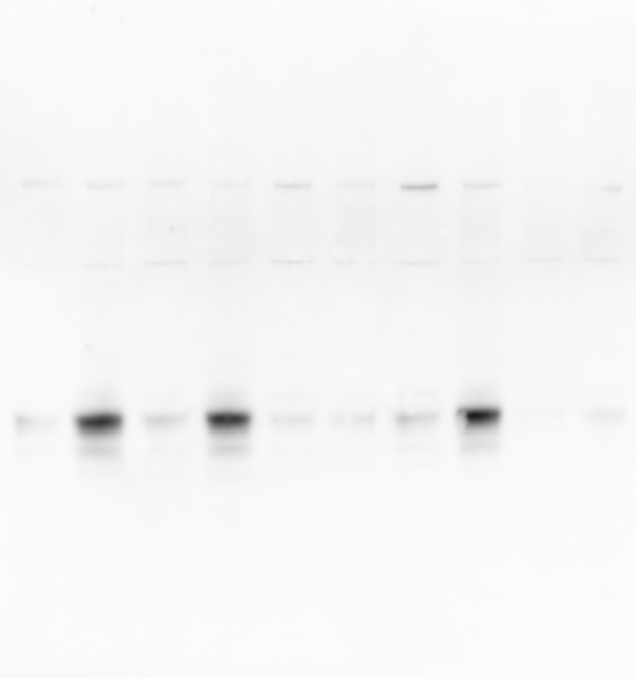

Supplement: Figure 2—source data 2. [file elife-102434-fig2-data2.zip › Fig2-sourcedata2/Jaber-sourcedata2-Fig2A-mdm2.jpg]

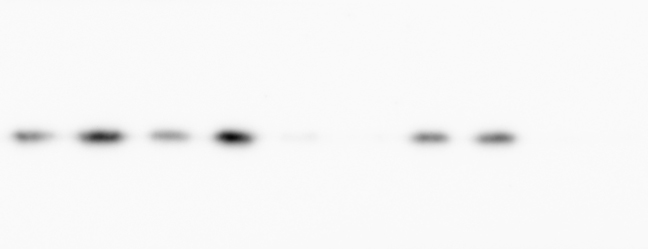

Supplement: Figure 2—source data 2. [file elife-102434-fig2-data2.zip › Fig2-sourcedata2/Jaber-sourcedata2-Fig2A-p21.jpg]

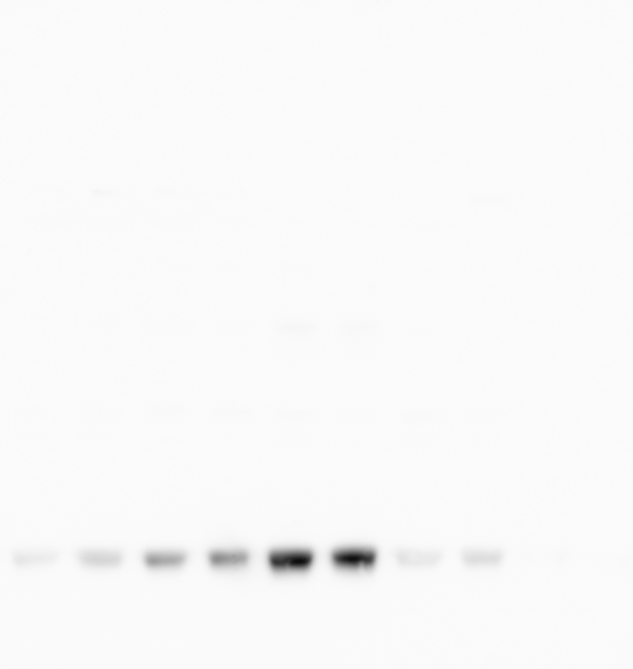

Supplement: Figure 2—source data 2. [file elife-102434-fig2-data2.zip › Fig2-sourcedata2/Jaber-sourcedata2-Fig2A-p53.jpg]

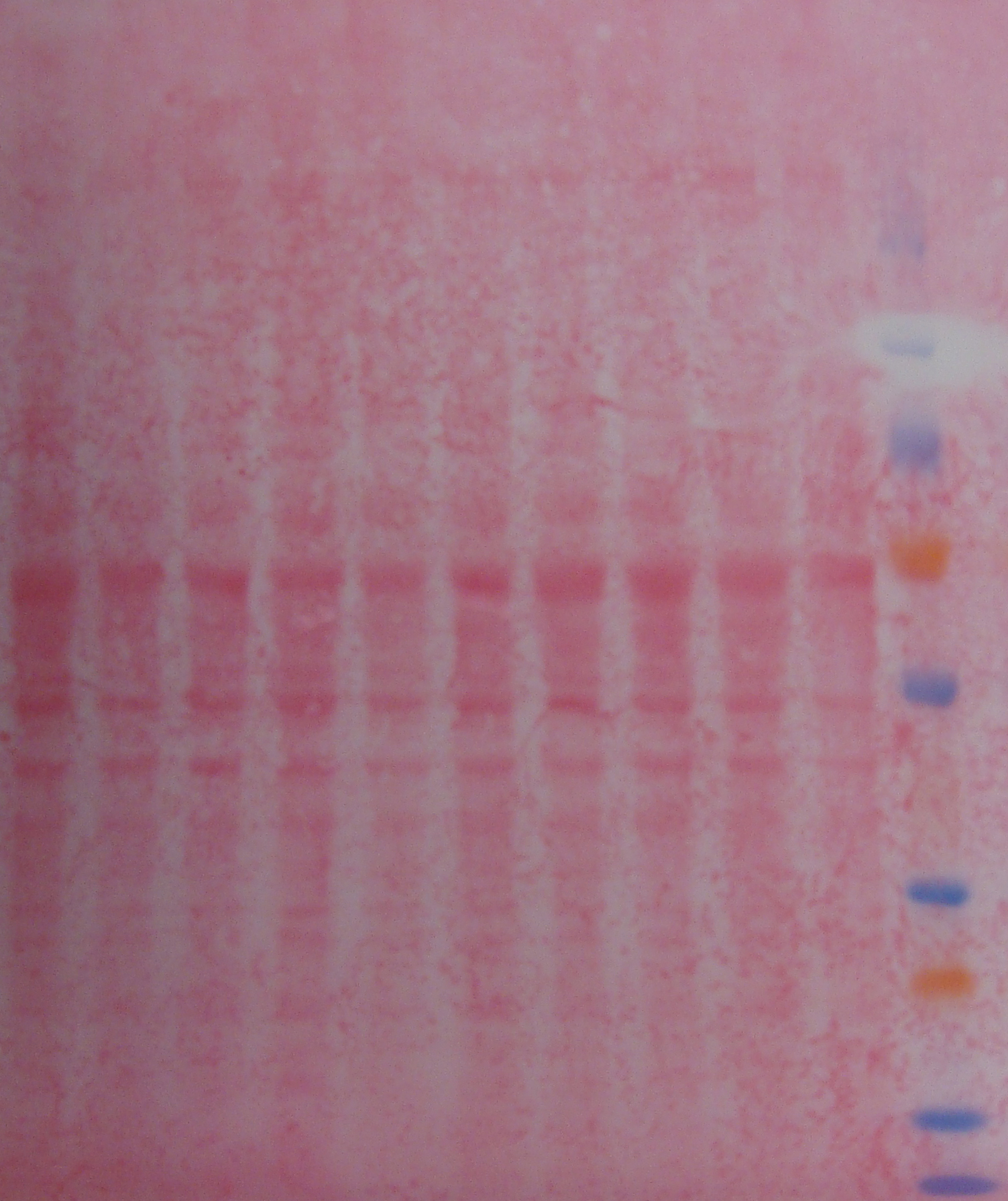

Supplement: Figure 2—source data 2. [file elife-102434-fig2-data2.zip › Fig2-sourcedata2/Jaber-sourcedata2-Fig2A-ponceau.jpg]

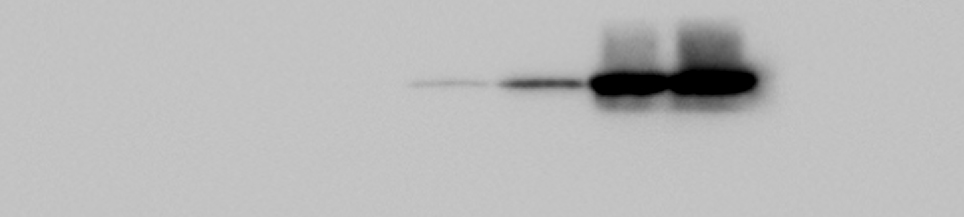

Supplement: Figure 2—source data 2. [file elife-102434-fig2-data2.zip › Fig2-sourcedata2/Jaber-sourcedata2-Fig2C-WT-H3.jpg]

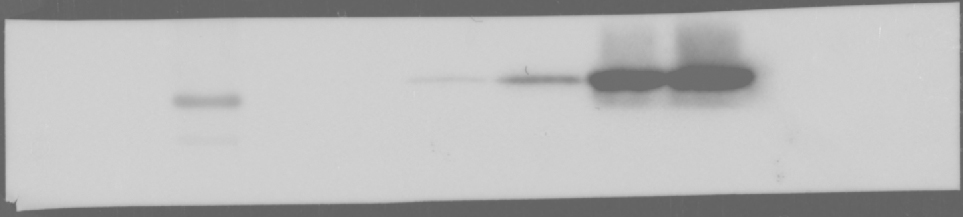

Supplement: Figure 2—source data 2. [file elife-102434-fig2-data2.zip › Fig2-sourcedata2/Jaber-sourcedata2-Fig2C-WT-H3+MW.jpg]

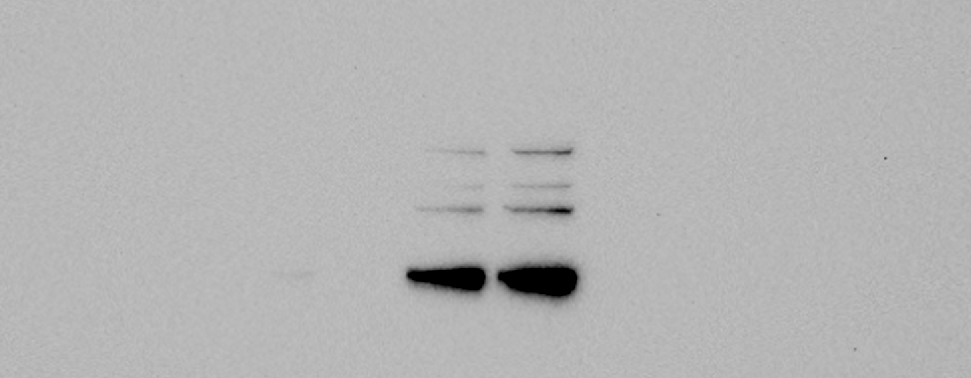

Supplement: Figure 2—source data 2. [file elife-102434-fig2-data2.zip › Fig2-sourcedata2/Jaber-sourcedata2-Fig2C-WT-Nup98.jpg]

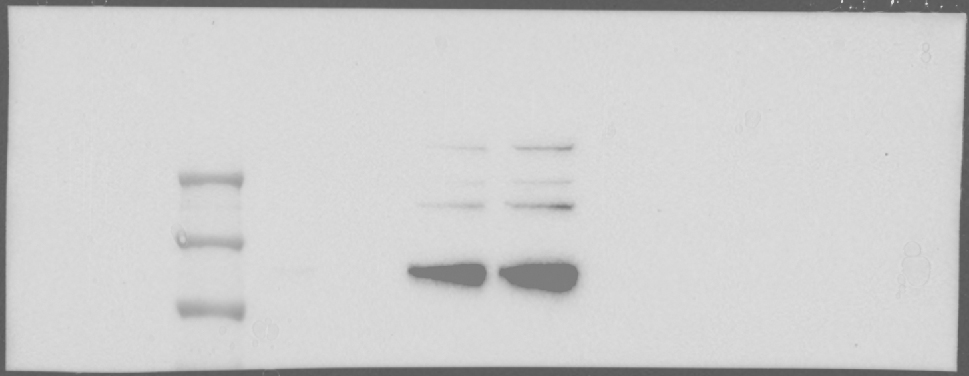

Supplement: Figure 2—source data 2. [file elife-102434-fig2-data2.zip › Fig2-sourcedata2/Jaber-sourcedata2-Fig2C-WT-Nup98+MW.jpg]

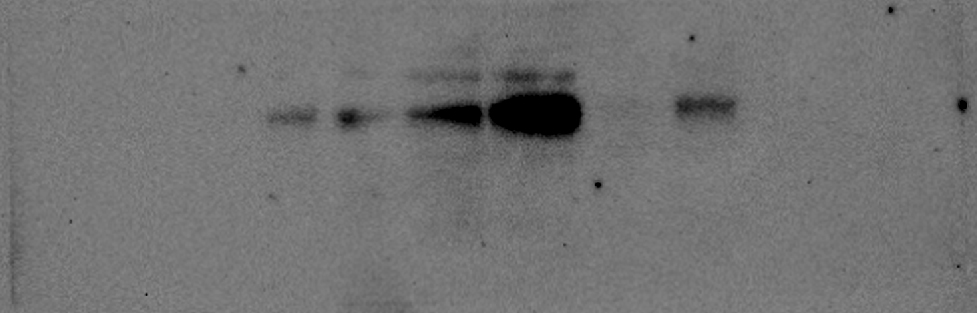

Supplement: Figure 2—source data 2. [file elife-102434-fig2-data2.zip › Fig2-sourcedata2/Jaber-sourcedata2-Fig2C-WT-p53.jpg]

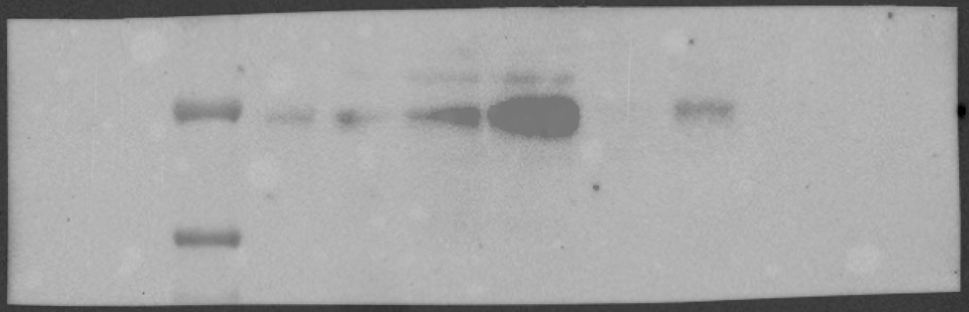

Supplement: Figure 2—source data 2. [file elife-102434-fig2-data2.zip › Fig2-sourcedata2/Jaber-sourcedata2-Fig2C-WT-p53+MW.jpg]

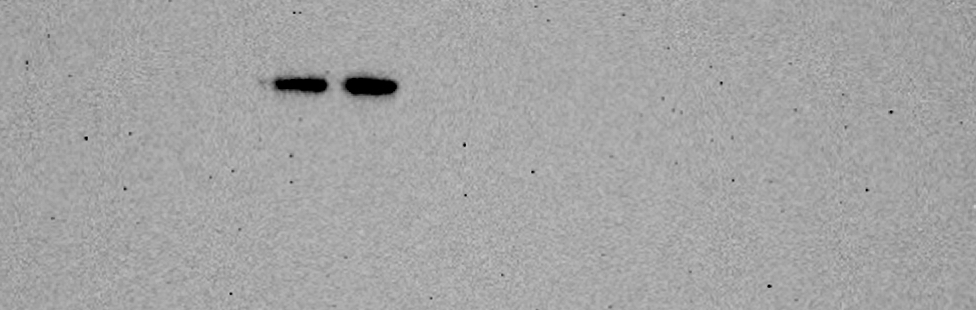

Supplement: Figure 2—source data 2. [file elife-102434-fig2-data2.zip › Fig2-sourcedata2/Jaber-sourcedata2-Fig2C-WT-tubulin.jpg]

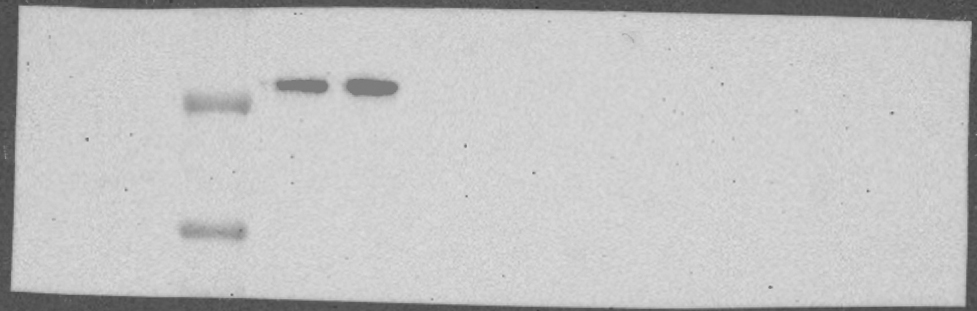

Supplement: Figure 2—source data 2. [file elife-102434-fig2-data2.zip › Fig2-sourcedata2/Jaber-sourcedata2-Fig2C-WT-tubulin+MW.jpg]

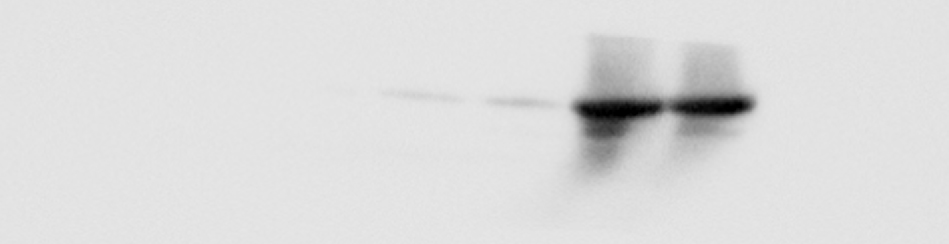

Supplement: Figure 2—source data 2. [file elife-102434-fig2-data2.zip › Fig2-sourcedata2/Jaber-sourcedata2-Fig2C-YC-H3.jpg]

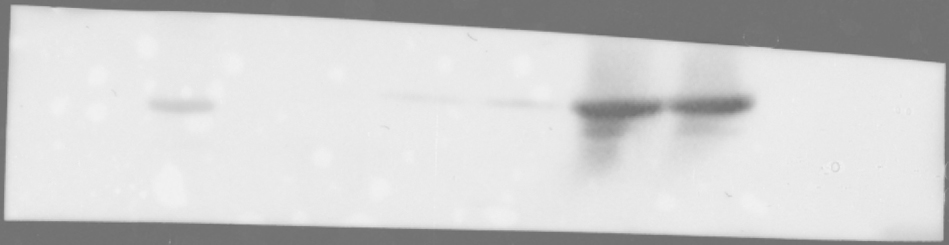

Supplement: Figure 2—source data 2. [file elife-102434-fig2-data2.zip › Fig2-sourcedata2/Jaber-sourcedata2-Fig2C-YC-H3+MW.jpg]

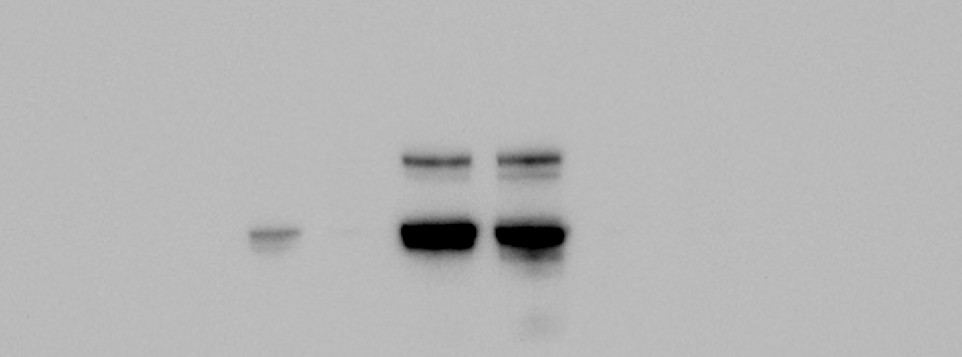

Supplement: Figure 2—source data 2. [file elife-102434-fig2-data2.zip › Fig2-sourcedata2/Jaber-sourcedata2-Fig2C-YC-Nup98.jpg]

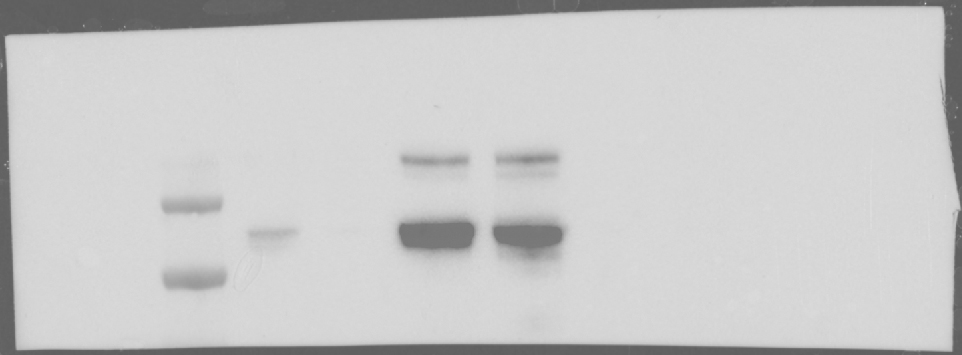

Supplement: Figure 2—source data 2. [file elife-102434-fig2-data2.zip › Fig2-sourcedata2/Jaber-sourcedata2-Fig2C-YC-Nup98+MW.jpg]

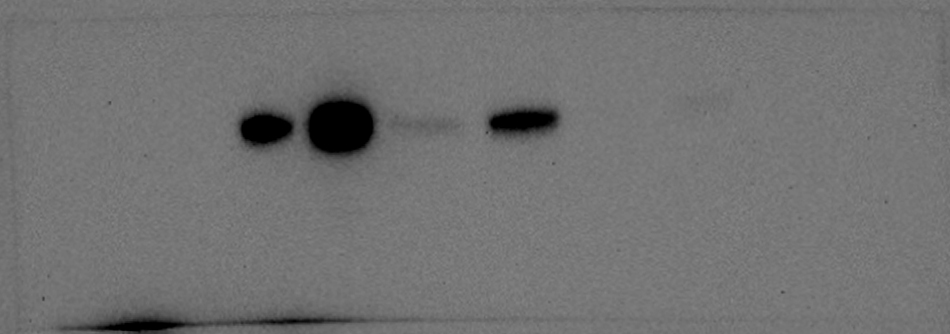

Supplement: Figure 2—source data 2. [file elife-102434-fig2-data2.zip › Fig2-sourcedata2/Jaber-sourcedata2-Fig2C-YC-p53.jpg]

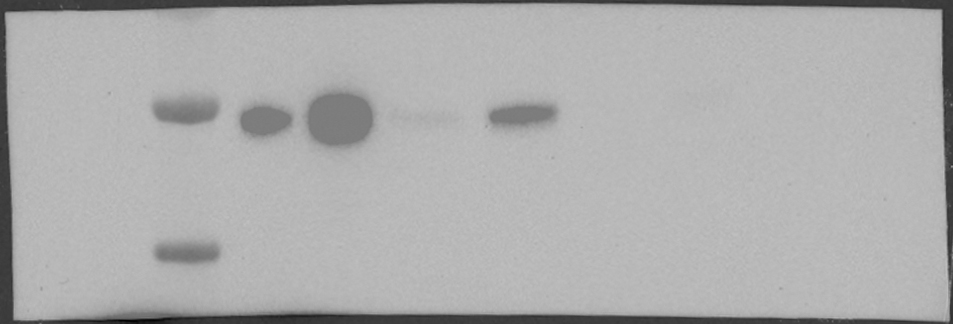

Supplement: Figure 2—source data 2. [file elife-102434-fig2-data2.zip › Fig2-sourcedata2/Jaber-sourcedata2-Fig2C-YC-p53+MW.jpg]

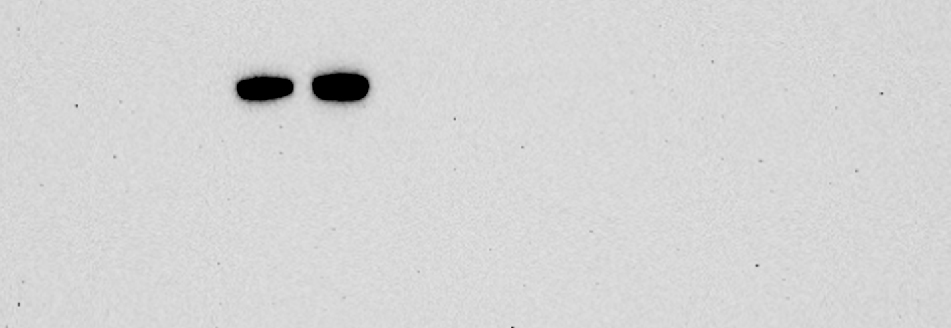

Supplement: Figure 2—source data 2. [file elife-102434-fig2-data2.zip › Fig2-sourcedata2/Jaber-sourcedata2-Fig2C-YC-tubulin.jpg]

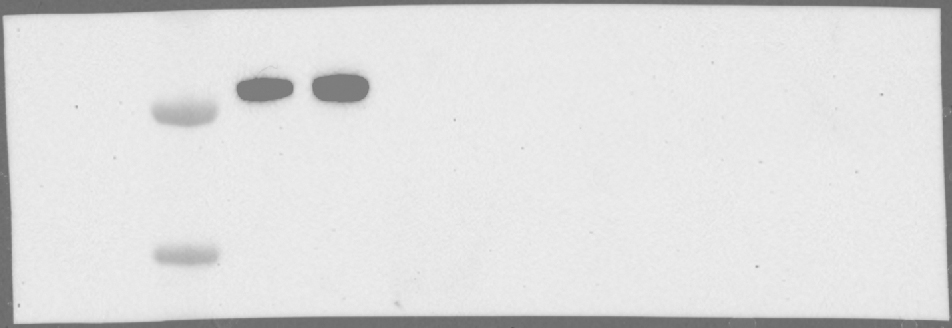

Supplement: Figure 2—source data 2. [file elife-102434-fig2-data2.zip › Fig2-sourcedata2/Jaber-sourcedata2-Fig2C-YC-tubulin+MW.jpg]
