# Supplementary material for: Oncogenic and teratogenic effects of Trp53Y217C, an inflammation-prone mouse model of the human hotspot mutant TP53Y220C
Source: eLife. 2025 Apr 14;13:RP102434. doi: 10.7554/eLife.102434 (PMC11996178; doi:10.7554/eLife.102434)
Supplement: Supplementary file 5. [file elife-102434-supp5.docx]

| **Locus** | **Forward Primer** | **Reverse Primer** | **Purpose** |
| --- | --- | --- | --- |
| *Trp53* | GCGTAGTGGTACCTTGTGAGCCCCCCGAGGTCT | ACCTCGGGGGGCTCACAAGGTACCACTACGCTG | Mutagenesis (YC-F/R) |
| *Trp53* | CCTGGGAATACTTCAAGAGACG | CGTGATATTGCTGAAGAGCTTG | Genotyping (a, b) |
| *Trp53* | GTGTGTTGGCCATCTCTGTG | AACCGGACTCAGCGTCTCTA | Genotyping (c, d) |
| *Ccl17* | GCTGGTATAAGACCTCAGTGGAGTGT | GCTTGCCCTGGACAGTCAGA | qPCR |
| *Ccl9* | GCACAGCAAGGGCTTGAAA | CAGGCAGCAATCTGAAGAGTCTT | qPCR |
| *Ccr3* | CTGGACTCATAAAGGACTTAGCAAAA | TCTTGATTTCATCTGTGTTGAATGC | qPCR |
| *Cdkn1a* | GCAGACCAGCCTGACAGATTTC | TTCAGGGTTTTCTCTTGCAGAAG | qPCR |
| *Cxcl10* | AATGAAAGCGTTTAGCCAAAAAA | GGTGTGTGCGTGGCTTCAC | qPCR |
| *Maob* | GGTACAACCAGAATCATCTCAACAA | CTTGACCAGATCCACCAATAAATTT | qPCR |
| *Mdm2* | GTCTACCGAGGGTGCTGCAA | AAGCAATGGTTTTGGTCTAACCA | qPCR |
| *Pls3* | CCAGCACTAACTAAACCTGAAAACC | GGTTCTTTCTTCCCGAGTTTCC | qPCR |
| *S100a8* | TCCTTTGTCAGCTCCGTCTTC | GACGGCATTGTCACGAAAGAT | qPCR |
| *S100a9* | ACAAATGGTGGAAGCACAGTTG | TCATTTATGAGGGCTTCATTTCTCT | qPCR |
| *Trp53* | AAAGGATGCCCATGCTACAGA | TCTTGGTCTTCAGGTAGCTGGAG | qPCR |
| *Usp9x* | CTTGCTCAGCAGATTTCTGATGA | GCTGTAATCCTCCACATCCTGAT | qPCR |
| *Ppia* | TCTCCTTCGAGCTGTTTGCA | CAGTGCTCAGAGCTCGAAAGTTT | qPCR (control) |
| *Rplp-0* | CGACCTGGAAGTCCAACTAC | ATCTGCTGCATCTGCTTG | qPCR (control) |
| *Cdkn1a* | GTGAGGAGGAGCATGAATGGA | GGCAAAGTGGGACGTCCTTA | ChIP |
| *Mdm2* | CCGAGTTGACTCAGCTCTTCCT | ACCCCAGCTGGAGACATGTC | ChIP |
| *Rbl2* | CCAAGGGAAGCCACATGGT | ACCAGCCTTTCCATCTGCAT | ChIP (control) |
